# c-JUN n-Terminal Kinase (JNK) Signaling in Autosomal Dominant Polycystic Kidney Disease

**DOI:** 10.33696/Signaling.3.068

**Published:** 2022

**Authors:** Abigail O. Smith, Julie A. Jonassen, Kenley M. Preval, Roger J. Davis, Gregory J. Pazour

**Affiliations:** 1 Program in Molecular Medicine, University of Massachusetts Medical School, Biotech II, Suite 213, 373 Plantation Street, Worcester, MA, USA 01605; 2 Department of Microbiology and Physiological Systems, University of Massachusetts Medical School, 55 Lake Avenue North, Worcester, MA, USA 01655

**Keywords:** Polycystic kidney disease, Jun N Terminal kinase, Polycystin-1, Polycystin-2, Cilia, Mus musculus, Mitogen-activated protein kinase signaling

## Abstract

Polycystic kidney disease is an inherited degenerative disease in which the uriniferous tubules are replaced by expanding fluid-filled cysts that ultimately destroy organ function. Autosomal dominant polycystic kidney disease (ADPKD) is the most common form, afflicting approximately 1 in 1,000 people and is caused by mutations in the transmembrane proteins polycystin-1 (Pkd1) and polycystin-2 (Pkd2). The mechanisms by which polycystin mutations induce cyst formation are not well understood, however pro-proliferative signaling must be involved for tubule epithelial cell number to increase over time. We recently found that the stress-activated mitogen-activated protein kinase (MAPK) pathway c-Jun N-terminal kinase (JNK) pathway is activated in cystic disease and genetically removing JNK reduces cyst growth driven by a loss of Pkd2. This review covers the current state of knowledge of signaling in ADPKD with an emphasis on the JNK pathway.

## Overview and Prevalence of Autosomal Dominant Polycystic Kidney Disease

Autosomal dominant polycystic kidney disease (ADPKD) is a common inherited disorder characterized by slow-growing, fluid-filled cysts in both kidneys. Liver and pancreatic cysts as well as cardiac and vascular abnormalities variably occur [1,2]. ADPKD symptoms include hypertension, kidney pain, hematuria, cyst infection, and urinary tract infections [1]. Over time, deteriorating kidney function leads to end stage renal disease (ESRD). While most ADPKD patients progress to ESRD by age 70, many reach it at younger ages [3]. Despite recent advances in therapy, ADPKD remains incurable, and for most patients treatment is limited to symptom management [4]. For patients with ESRD, renal replacement therapy in the form of dialysis or transplant is inevitable. Unfortunately, renal transplantation does not cure ESRD and patients commonly experience acute rejection, cardiovascular diseases, infection, and malignancy [5].

ADPKD is the most common inherited kidney disease, afflicting approximately 1 in 1,000 people in the US. Prevalence was originally estimated by postmortem kidney cyst detection [6,7] but is supported by recent large-scale population sequencing where 0.93 pathogenic mutations were found per 1,000 people [8]. In contrast, healthcare databases report 0.43 ADPKD cases per 1,000 people in the US [9,10]. This suggests significant underdiagnosis likely due to late onset and variable clinical presentation. Nevertheless, as the fourth leading cause of ESRD in the US, ADPKD places a heavy burden on individuals and healthcare systems alike [11].

## Polycystin-1 and Polycystin-2 Mutations are the Major Cause of PKD

Genetic mutations in either polycystin-1 (*Pkd1)* or polycystin-2 (*Pkd2*), comprise most ADPKD cases. *Pkd1* mutations make up 78% of cases, *Pkd2* mutations make up 15%. The remaining cases are due to unidentified or rare mutations [12], including *DNAJB11* [13], *GANAB* [14], *ALG9* [15], and *IFT140* [16]. *Pkd1* and *Pkd2* phenotypes are qualitatively similar, but *Pkd1* mutations typically cause more severe disease with *Pkd1* patients progressing to ESRD twenty years earlier than *Pkd2* patients [17–19].

*Pkd1* is located on chromosome 16p13.3 within a region of highly homologous repeats [20,21] where it encodes a large integral membrane protein of 4,304 amino acids. The N-terminal extracellular domain contains multiple domains with putative roles in cell adhesion, followed by 11 transmembrane passes and a cytoplasmic C-terminus [20]. Pkd1 is widely expressed in different tissues, with higher levels during development than adulthood [22]. It localizes to the plasma membrane and is concentrated in primary cilia, antennae-like sensory organelles that project from the apical cell membrane [23–25]. Pkd1’s physiological contexts remains obscure although it has been hypothesized to function as an atypical adhesion G protein-coupled receptor (GPCR) [26] or a Wnt ligand receptor [27].

*Pkd2* maps to chromosome 4q22.1 [28,29]. The protein, which is a member of the Trp channel family, consists of 968 amino acids with intracellular N- and C-terminal regions and 6 transmembrane passes. Pkd2 is ubiquitously expressed and localizes to the plasma membrane, ciliary membrane, and endoplasmic reticulum [25,30,31]. Pkd2 is a calcium-permeable non-selective cation channel [32–34] with activity in the cilium [35,36].

Pkd1 complexes with Pkd2 [37–41] as a heterotetrameric complex consisting of three Pkd2 molecules and one Pkd1 molecule. Oligomerization appears to depend on the extracellular polycystin domains shared by both molecules [42], although earlier studies found that Pkd1 and Pkd2 interact via intracellular C-terminal coiled-coiled domains [37]. The ciliary polycystin complex maintains tubule architecture through an undefined mechanism. Leading theories include fluid flow sensation [43] and ligand detection in the filtrate [27].

To a limited extent, genotype predicts the clinical course of ADPKD. At a particular age, *Pkd1* patients have greater total kidney volume than *Pkd2* patients, indicating greater cystic load. However, cyst growth rate over time is the same in *Pkd1* and *Pkd2* patient populations, suggesting that the difference in disease progression is due to cyst initiation [44]. More than 2,000 *Pkd1* mutation variants and more than 250 variants of *Pkd2* have been identified [http://pkdb.pkdcure.org]. Predicted truncations are associated with the most severe disease outcomes, while missense mutations produce milder disease [18,45,46]. This is consistent with research showing that functional Pkd1 protein levels correlate with disease severity [47].

## Cyst Initiation Theories

**Figure 1:**
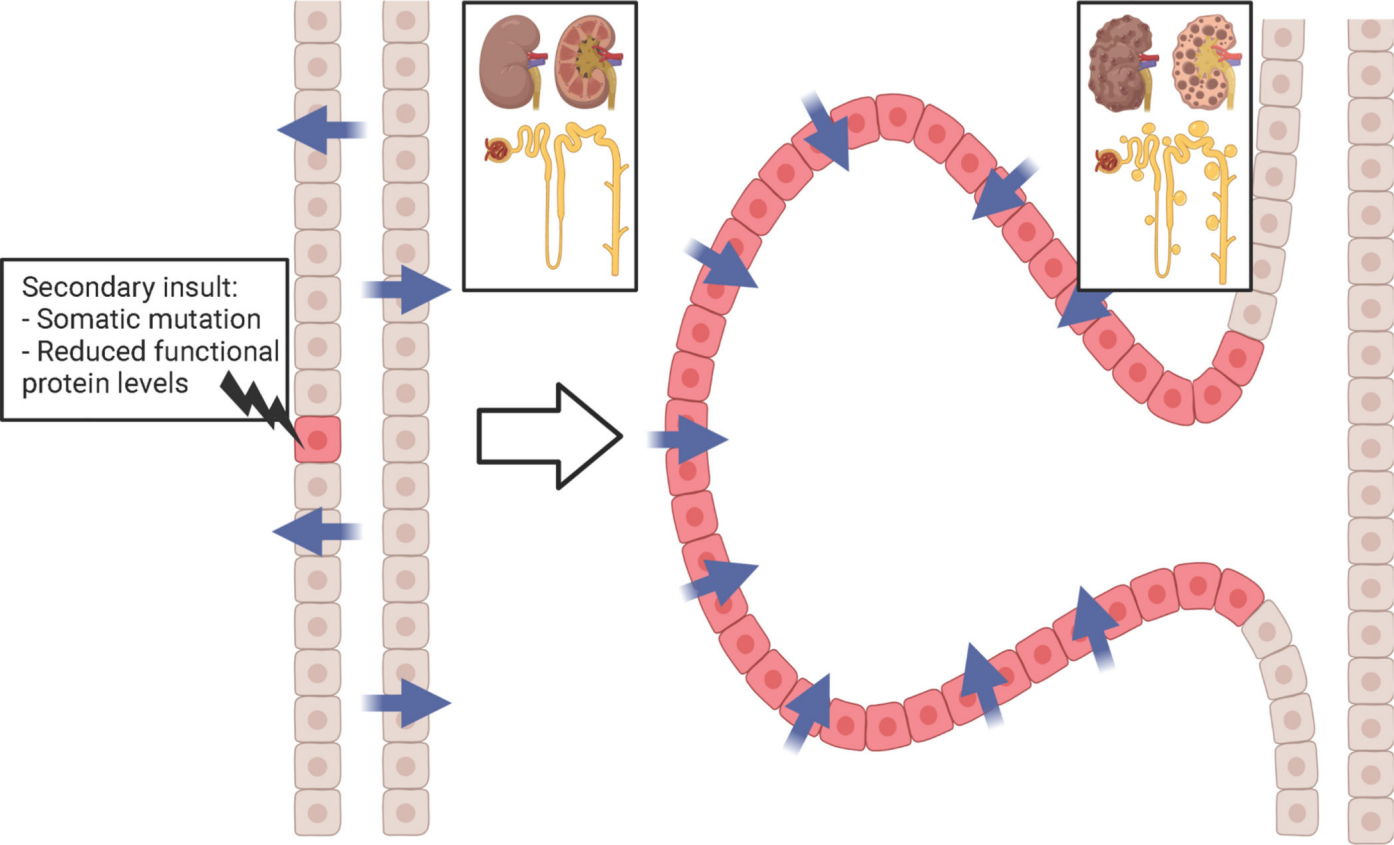
Cyst initiation in ADPKD requires a secondary insult. Although ADPKD is dominantly inherited, cyst initiation is recessive at the cellular level. Cyst initiation occurs when tubule epithelial cells harboring one mutated allele of Pkd1 or Pkd2 receive a secondary insult, either by spontaneous somatic mutation of the healthy allele (two-hit hypothesis) or by any mechanism that reduces the function of the polycystin proteins below a critical threshold (threshold model). Created with BioRender.com

A person with ADPKD carries the pathogenic mutation in every cell, but cysts develop focally along kidney tubules. As disease progresses, cysts increase in both size and number. What triggers individual cells to switch from normal to cystic phenotype? Analyses revealed that epithelial cells lining human cysts are clones harboring the same somatic mutation in trans with the inherited mutation [48]. These findings suggest that cyst initiation occurs when a tubule cell with one mutant polycystin allele loses its remaining functional copy. The loss of both copies triggers focal proliferation and cyst formation. Analogous to Knudson’s two-hit hypothesis of tumor progression [49], this theory of cyst initiation is known as the two-hit model of ADPKD progression (Figure 1) [50,51].

Additional evidence for the two-hit model comes from mouse genetic studies. *Pkd2*^*−/−*^ mice die after embryonic day 14.5 with cardiac malformations, impaired right-left symmetry, and cysts in the kidney, liver, and pancreas [52]. *Pkd1*^*−/−*^ mice also die *in utero* with massively cystic kidneys and pancreas, and their lungs are underdeveloped likely due to kidney malfunction causing oligohydramnios [53,54]. Early postnatal deletion of both alleles of *Pkd1* or *Pkd2* circumvents the embryonic lethality but still causes rapid cystic disease progression [55]. In sharp contrast to homozygous knockout mice, heterozygous mutations do not perturb normal development, lifespan, or produce significant cystic disease [47,56,57]. Wu et al. showed that increasing the somatic mutation rate in *Pkd2*^*+/−*^ by introducing an unstable *Pkd2* allele greatly increased cyst progression [56]. This evidence supports the idea that tubule cells function normally with one allele of *Pkd1* or *Pkd2*, and only with a “second hit” do cysts form.

**Figure 2:**
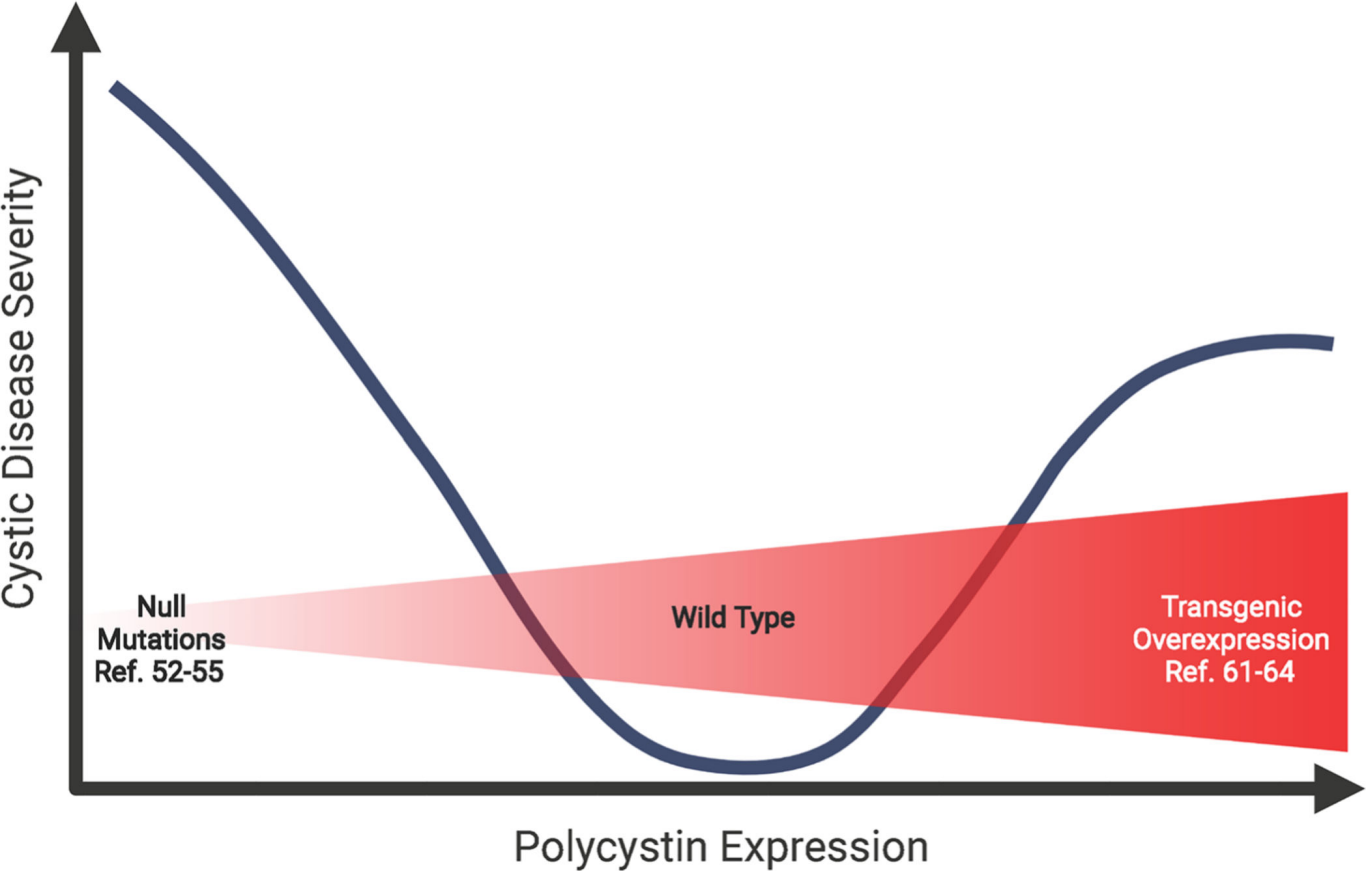
Threshold model of cyst initiation incorporates cystic disease caused by polycystin overexpression. Mice completely lacking Pkd1 or Pkd2 from conception demonstrate the most severe disease (embryonic lethality). Conditional homozygous deletion either postnatally or with tissue-specificity also induces severe disease with early mortality. Heterozygous mutants exhibit milder disease, suggesting that the closer to the wild-type range of gene expression, the milder the disease phenotype. Interestingly, expressing higher than normal levels of the polycystins has also been shown to cause cystic disease. The figure depicts a “threshold model” or dose-dependent model for developing cystic disease. Created with BioRender.com

Scenarios beyond the two-hit model, such as hypomorphic mutations, compound heterozygosity, and polycystin overexpression, require alternative modes of cyst initiation. The dosage threshold model hypothesizes that epithelial cells function normally only when polycystin levels are within a limited range. Above or below that range, cells exhibit an abnormal cystic phenotype (Figure 2). Lantinga-van Leeuwen *et al.* provided evidence for the threshold model by inserting a neomycin cassette in a *Pkd1* intron to create a hypomorphic allele. This insertion disrupted splicing and reduced the amount of correctly spliced *Pkd1* to 15% of normal. Unlike mice homozygous for true null mutations, the hypomorphs survived development but developed significant cystic kidney disease by one month of age [47]. To further explore how *Pkd1* dosage affects cyst formation, researchers used a hypomorphic mutation based on human disease variant p.R3277C (*Pkd1*^*RC*^). Like *Pkd1*^*+/+*^ mice, *Pkd1*^*RC/+*^ mice did not develop cysts within one year. *Pkd1*^*RC/RC*^ mice were viable with slow disease progression and no change in survival compared to wild type. *Pkd1*^*RC/-*^ mice were also viable but rapidly developed severe cystic disease associated with premature death. This contrasts with *Pkd1*^*−/−*^ mice, which die *in utero* [58]. *Pkd2*^*nf3*^ hypomorphic mice exhibited similar results [59]. Furthermore, transgenic *Pkd2* expression rescued the embryonic lethality phenotype in *Pkd2*^*−/−*^ mice. *Pkd2* transgene expression dose-dependently slowed cystic phenotype progression [60]. The findings show that functional polycystin protein dose inversely correlates with disease progression and severity.

Paradoxically, overexpressing polycystins also induces cysts. In a rescue experiment, overexpressing human *Pkd1* prevented embryonic lethality in *Pkd1*^*−/−*^ mice but caused cystic kidney disease in *Pkd1*^*+/+*^ animals [61]. Subsequent models confirmed that *Pkd1* overexpression [62,63] and, to a lesser extent, *Pkd2* [64] cause cystic kidney disease. While most ADPKD cases involve loss-of-function mutations, high Pkd1 and Pkd2 levels have been detected in human cystic renal tissues [22,65]. While it is not known how overexpressed polycystins can cause cyst formation, it is possible that abnormally high levels of one of the two polycystins may disrupt the integrity of the polycystin complex, leading to loss of function.

Interestingly, mice homozygous for the *Pkd1*^*RC*^ mutation developed focal cysts even though every cell in the tubule was genetically identical [58]. This suggests that additional stimuli are needed to convert Pkd1-deficient tubule epithelium into cystic epithelium. The additional stimuli, called the third hit, was proposed when it was found that deletions of *Pkd1* or *Ift88* in mice with fully developed kidneys were largely immune to cyst formation [66–68] unless they incurred kidney injury [69]. Acute kidney injury (AKI) represents the extreme end of potential renal challenges that ADPKD patients face and can occur due to hypovolemia, acute urine outflow obstruction, or nephrotoxic drug response. Studies in animal models show that cyst growth increases after AKI [70–72]. AKI also activates c-Jun N-terminal kinase (JNK) in animal studies and human tissue samples, and JNK inhibition reduces organ damage due to acute injury [73–77]. As discussed below, JNK appears to promote cyst growth in ADPKD by amplifying cellular response to injury.

## Primary Cilia are Critical to Preventing Cystic Disease

The epithelial cells that form cysts in ADPKD each possess a single primary cilium projecting into the tubule lumen. Primary cilia are microtubule-supported, membrane-covered organelles that function as signal transduction centers for the cell [78,79]. Cilia mutations underlie syndromic forms of polycystic kidney disease [80], including Bardet-Biedl syndrome [81], Nephronophthisis [82,83], Joubert syndrome [84], and Meckel-Gruber syndrome [85, 86]. Mice with mutations in ciliary assembly genes (*Kif3a, Ift88, Ift140, Ift20*) develop renal cysts [66,80,87,88]. Pkd1 and Pkd2 co-localize in primary cilia membranes [25,31].

Accumulating evidence points to a requirement for ciliary localization of polycystins to prevent cyst formation [89–92]. Cai *et al.* identified missense mutations in *Pkd1* and *Pkd2* that impaired trafficking to cilia while preserving protein levels and other aspects of protein function such as the Pkd1-Pkd2 interaction. These trafficking mutations failed to rescue *Pkd1*^*−/−*^ lethality or cystic disease caused by kidney-specific *Pkd1* deletion [91]. In a similar study, Walker et al. studied the *Pkd2*^*lrm4/lrm4*^ mouse line, which carries a missense mutation in Pkd2 that preserves its channel properties but disrupts its ability to localize to cilia. Pkd2 levels in *Pkd2*^*lrm4/lrm4*^ embryonic kidneys were equivalent to *Pkd2*^*+/−*^ mice, which do not develop severe disease. In contrast the *Pkd2*^*lrm4/lrm4*^ died *in utero* with kidney cysts similar to those observed in *Pkd2*^*−/−*^ mice. They attributed the cystic phenotype to the failure of *Pkd2* to localize to cilia in the mutant mice [90]. To strengthen the conclusions made by both studies, future experiments should retarget the mutant forms of Pkd1 and Pkd2 to the ciliary compartment to determine if relocalization rescues the cystic phenotype.

Interpreting results from point mutations affecting polycystin trafficking is challenging because these mutations may affect other functions. An alternative is to disrupt the delivery of wild-type polycystins to cilia. Two approaches have been taken in this space. One focuses on the role *Tulp3* plays in cystic disease [89,93]. *Tulp3* is an intraflagellar transport associated protein necessary for trafficking transmembrane proteins including Pkd1 and Pkd2 to cilia, but its deletion does not alter cilia structure or whole cell Pkd1 or Pkd2 levels [94]. *Tulp3* deletion caused polycystic kidney disease that was intermediate between *Pkd1* deletion and cilia deletion, implying that trafficking to cilia is a critical factor in the ability of polycystins to prevent cyst formation. The second approach focuses on Bardet-Biedl syndrome (BBS) proteins that bind to the polycystins and deliver Pkd1 to cilia [95]. The observation that BBS patients develop cystic disease supports the idea that the polycystins must be trafficked to and enter primary cilia. However, a caveat to both the Tulp3 and BBS studies is that these proteins play roles in cilia biology beyond the polycystins, and these other functions could be important to prevent cystic disease.

Experiments involving animals lacking cilia and *Pkd1* or *Pkd2* revealed a complex relationship between cilia and polycystin signaling [92]. Mice with double mutations in *Pkd1* and *Kif3a* or in *Pkd2* and *Ift20* exhibited an intermediate phenotype between the milder cilia-induced and more severe polycystin-induced cystic kidneys. Furthermore, disease severity positively correlated with the time between polycystin removal and cilia disappearance. Cilia disruption reduced tubule epithelial cell proliferation in polycystin mutants. These findings lead to the hypothesis that cyst growth depends on propagating a pro-proliferative signal by intact cilia, which was termed the cilia-dependent cyst-activating (CDCA) signal. When Pkd1 and Pkd2 are present, they inhibit the CDCA, thereby inhibiting tubule cells from proliferating and forming cysts. The model predicts that CDCA activity would be low in control kidneys, greatly elevated in Pkd mutant kidneys, and brought back towards controls in Pkd and cilia double mutant kidneys. ERK, mTOR, and cAMP signaling were tested as candidates for propagating the CDCA signal. However, none of these pathways matched the activity pattern predicted, leaving the identity of the CDCA unknown [92]. To identify candidate pathways controlling the CDCA, transcription profiling was used to identify genes whose expression is elevated by the loss of *Pkd2* and brought back towards controls in *Pkd2*, *Ift88* double mutant kidneys. Genes that matched the CDCA profile were enriched in cell proliferation functions. Cdk1 stood out as a strong candidate as its expression was increased ~5 fold by the loss of *Pkd2* and returned to normal in the *Pkd2* cilia-minus kidneys. Importantly, the loss of Cdk1 reduced cyst growth in a *Pkd1* mutant kidney, making the *Cdk1* pathway a strong candidate for the CDCA [96].

## Signal Transduction by the Polycystin Complex

The physiological purpose of ciliary-localized polycystins is not known. One popular model posits that polycystins function as mechanosensors for fluid flow through the tubule lumen. This idea grew from studies showing that intracellular calcium increased in response to cilia deflection [97]. Comparing calcium responses to shear stress in collecting duct cells from wild type and homozygous null *Pkd1 (Pkd1*^*del34/del34*^*)* mice found that cytosolic calcium was transiently increased in wild type cells, but not *Pkd1* mutant cells. Blocking antibodies to Pkd2 also abolished the flow-induced calcium signal [43]. These findings supported a model in which polycystin mutations disrupt mechanosensation and dysregulate calcium signaling, leading to abnormal downstream signaling and cysts. However, the results left open the possibility that calcium changes start in the cytosol rather than the cilium of kidney tubule cells. To clarify the source of the initial calcium influx, Delling *et al.* examined collecting duct cells from a transgenic mouse expressing a fluorescent calcium reporter specific for changes in the ciliary calcium levels. These cells did not exhibit any flow-induced calcium fluctuations under physiologically relevant flow conditions that caused cilia bending. Surprisingly, cilia bending did not elicit cilia-derived changes in calcium in any tissue tested. Instead, they reported that elevated calcium in the cytoplasm could diffuse into the cilium in a relatively short time frame of less than 200 milliseconds, potentially explaining the discrepancy with earlier findings. They also noted that extreme flow rates could tear cilia tips, allowing extracellular calcium to flow into the cilium. In conclusion, they suggested that cilia may function as mechanosensors, but not through a transient increase in calcium concentration [98].

Recently, an *intra vital* technique to visualize renal cilia in a live mouse showed that under normal conditions, the tubule luminal cilia do not oscillate but rather remain bent flat against the tubule wall [99]. This suggests that, at least in mice, cilia are typically subjected to maximum deflection. An important caveat to this work is that the resting heart rate in mice is 500–700 beats per minute compared to 50–70 beats per minute in humans. In mice near death, the cilia did oscillate as heart rate slowed [100]. The studies also observed that tubule epithelium undergoes dynamic cytoplasmic calcium fluctuations under normal conditions, but they did not observe any correlation between calcium changes and cilia bending. It will be interesting to use this live imaging approach to see if mice carrying cilia or polycystin mutations display disrupted calcium signaling.

Alternatively, the ciliary polycystin complex could be detecting molecules in the filtrate. For example, Wnt ligands bind Pkd1 to effect changes in calcium signaling [27]. In addition, structural similarities [101,102] and a capacity to bind heterotrimeric G proteins [103–105] suggest that Pkd1 may be an atypical adhesion GPCR.

## cAMP and Calcium Signaling Promote Cyst Growth

The fundamental factors underpinning cyst growth in ADPKD are tubule epithelial cell proliferation and fluid secretion. Healthy kidney tubules have a narrow lumen with a diameter of about 30 microns [106]. In contrast, cysts can reach over 70 millimeters in diameter [107]. Using scanning electron microscopy to directly count cells and measure their surface area, researchers determined that tubule cells proliferate rather than expand as the cystic lumen increases in surface area [108]. Microdissection and serial scanning electron microscopy showed that most cysts lacked connections to tubules suggesting that cyst fluid comes from transepithelial secretion rather than accumulated glomerular filtrate. Understanding what stimulates proliferative and secretory changes in cystic epithelium is critical to future therapy development.

**Figure 3:**
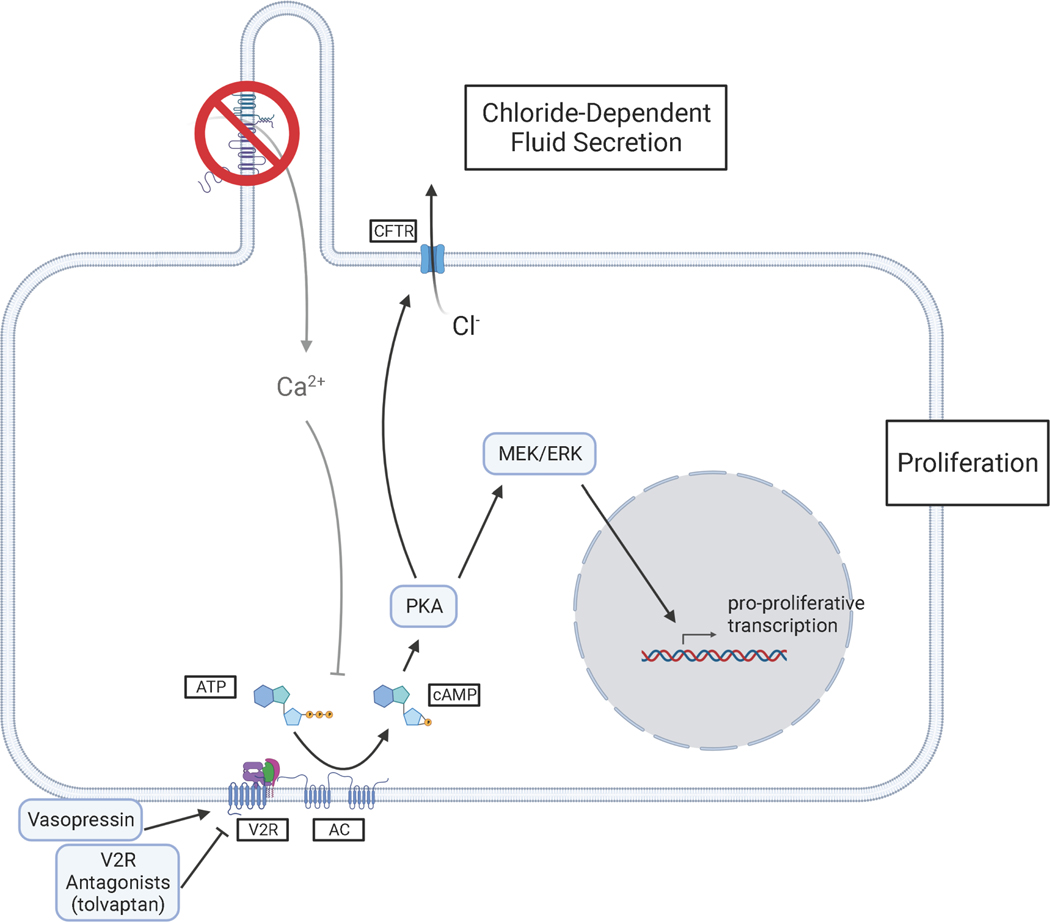
Disrupted calcium and cAMP signaling promotes cystic changes in tubule epithelial cells. Mutations in Pkd1 and Pkd2 in tubule epithelial cells result in lowered intracellular calcium, increased cAMP production, and altered sensitivity to cAMP. High intracellular cAMP levels promote fluid secretion via CFTR channels and increased proliferation via PKA/MEK/ERK. Fluid secretion and proliferation promote kidney cyst formation and expansion. Created with BioRender.com

The finding that cyst fluid and cystic tissues contained high cAMP levels led to the hypothesis that this ubiquitous second-messenger promotes cystic changes in tubule epithelium [109–111]. This idea is supported by *in vitro* studies showing that cAMP stimulated cyst growth in wild-type Madin-Darby Canine Kidney (MDCK) cells grown in a three-dimensional hydrated collagen matrix [112]. In control media, the cells formed small, slow-growing spheres with apical membranes toward the lumen. However, adding cAMP agonist prostaglandin-E1 to the media expanded the spheres into fluid-filled cysts [110]. Solute transport inhibitors blocked cyst expansion, suggesting that cyst growth depends on cAMP-stimulated chloride secretion. cAMP promotes secretion in tubule epithelial cells by activating cystic fibrosis transmembrane conductance regulator (CFTR) channels in the apical cell membrane (Figure 3) [113]. Further evidence that cyst growth depends on secretion comes from families with both cystic fibrosis and ADPKD. Patients with mutations in CFTR and PKD1 have less severe kidney and liver disease than patients with ADPKD alone [114,115].

cAMP’s role in proliferation was less clear. *In vitro* studies showed that cAMP inhibited proliferation by blocking the ERK pathway [116,117]. In addition, vasopressin, a hormone that increases intracellular cAMP levels, reduced ERK activation and proliferation in kidney cells derived from rat [118] and dog [119]. Rats treated with folic acid to induce rapid kidney cell proliferation experienced decreased proliferation rates upon cAMP treatment as measured by radioactive thymidine incorporation [120]. However, several studies showed that cyst fluid from human and mouse kidneys stimulated increased intracellular cAMP levels in cultured kidney cells and promoted secretion and proliferation. Specific cAMP agonists recapitulated secretion but had no significant effect on proliferation, suggesting that mitogens in the cyst fluid stimulated growth by additional mechanisms [121,122].

The experiments documenting cAMP inhibiting proliferation used wild-type cells, whereas the cells that form cysts in ADPKD are mutated in at least one allele of *Pkd1* or *Pkd2*. To determine how cAMP effects mutated cells, researchers obtained cystlining epithelial cell cultures from human ADPKD kidneys. Comparing these ADPKD cells to cells derived from healthy kidney cortex, they made the surprising discovery that ADPKD cells proliferated in response to cAMP while wild-type cells did not [123,124]. cAMP agonists enhanced ERK activity in ADPKD cells but not wild-type kidney cells [124].

Polycystin mutations are associated with reduced resting intracellular calcium levels [125]. Reducing calcium levels in wild-type cells using channel blockers switched their response to cAMP from quiescent to proliferative [126]. Calcium restriction also led to cAMP-dependent ERK activation (Figure 3). Transfecting wild-type cells with the C-terminus of Pkd1 also produced the proliferative response to cAMP, which was reversed by replenishing calcium levels by calcium ionophore treatment [126]. Importantly, elevating calcium levels in ADPKD cells induced normal response to cAMP and reduced cyst formation in an organoid model [125]. The pro-secretory and pro-proliferative effects on ADPKD cells suggested that reducing cAMP levels would be a viable option for slowing cyst growth.

Two important treatments to reduce cAMP in kidney tubule epithelial cells have been tested in pre-clinical and clinical trials. Somatostatin analogues reduce adenylyl cyclase activity by binding to somatostatin receptors on the cell surface, which reduces intracellular cAMP production [127,128]. Somatostatin analogues effectively reduced cyst growth in initial clinical trials [129]. However, subsequent larger randomized clinical trials showed that somatostatin analogues did not preserve kidney function enough to justify the risks [130]. The larger trials enrolled later stage ADPKD patients with significantly reduced kidney function at baseline and it is possible that a therapeutic effect may be seen if used in patients with milder disease. Notably, somatostatin analogues show promise in reducing liver cysts [131,132].

Healthy mammalian kidneys experience high tonic exposure to vasopressin to prevent excessive water loss. The hormone acts on vasopressin 2 receptors in the collecting ducts and distal nephron [133]. Vasopressin 2 receptor activates adenylyl cyclase 6 through G_s_ proteins, thereby increasing cAMP production [134]. Vasopressin 2 receptor antagonists such as tolvaptan prevent vasopressin from binding to vasopressin 2 receptor, which effectively decreases intracellular cAMP (Figure 3). Vasopressin 2 receptor antagonism reduced cyst growth in animal models [135,136] and human ADPKD cells in a collagen matrix [137]. Tolvaptan successfully reduced the rate of decline in kidney function in clinical trials and was approved for treating ADPKD in 2018.

## JNK is a MAP Kinase Pathway Activated by PKD

The mitogen-activated protein kinase (MAPK) superfamily includes the ERK1/2, p38, JNK, and ERK5 signaling pathways, all of which are activated through multi-tiered phosphorylation cascades in response to various stimuli [138]. MAPK signaling broadly impacts human health and disease due to its roles in cell proliferation, survival, and differentiation [139]. Our recent work showed that Pkd2 loss activates JNK signaling and that genetic reduction of JNK activity reduced cyst growth triggered by loss of Pkd2 [140].

**Figure 4:**
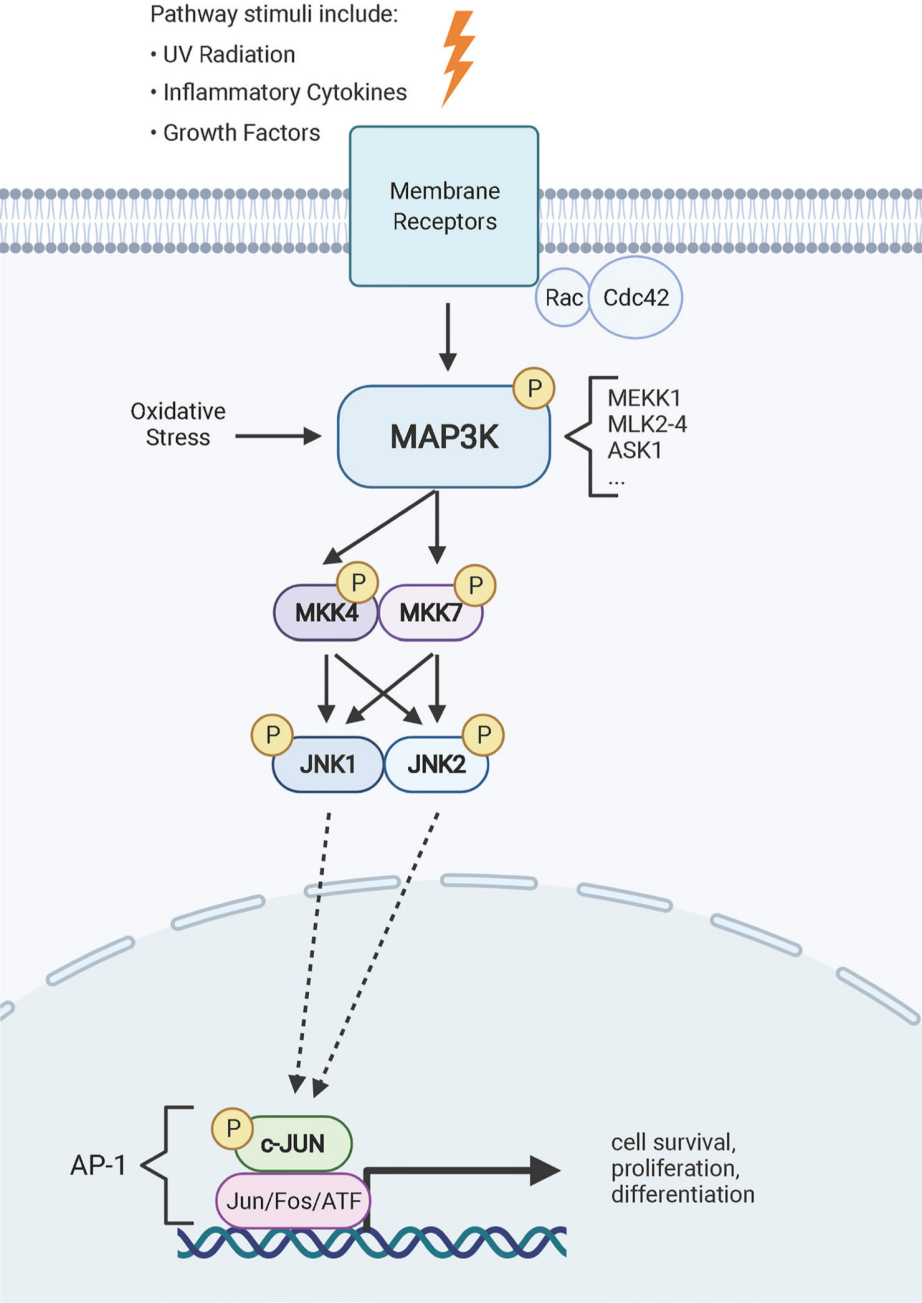
JNK is a stress-activated MAP kinase phosphorylation cascade. A variety of upstream stressors activate JNK through a phosphorylation cascade including MAP3Ks, MAP2Ks and JNKs. JNK1 and JNK2 are expressed ubiquitously including in the kidney and liver epithelium. Phosphorylated JNKs activate downstream targets such as AP-1 transcription factor subunits to control diverse cellular processes including proliferation, differentiation and cell survival. Created with BioRender.com

JNKs become activated by dual phosphorylation of threonine and tyrosine in the T-P-Y motif within the activation loop [141]. JNK phosphorylation is mediated by two MAP kinase kinases (MAP2K), MKK4 and MKK7. In turn, MAP2Ks are activated through phosphorylation by MAP kinase kinase kinases (MAP3K). The mouse and human genomes encode 24 MAP3Ks that feed into the ERK1/2, JNK, p38, and ERK5 pathways, with at least 14 known to phosphorylate MKK4 or MKK7 upstream of JNKs [142,143]. MAP3Ks are activated by various stimuli including cellular stress, inflammatory cytokines, growth factors, and GPCR agonists. These signals are typically detected by receptor tyrosine kinases, GPCRs, and other membrane receptors, and the signal is propagated to the MAP3Ks through the actions of Rac1 and Cdc42, although a variety of other mechanisms exist [144]. For example, reactive oxygen species activate the MAP3K, ASK1 through a mechanism involving thioredoxin binding [145]. It is thought that the specificity of JNK activation in different contexts is mediated by MAP3K activation (Figure 4).

Three highly homologous genes encode Jun N-terminal kinases: *JNK1/MAPK8*, *JNK2/MAPK9,* and *JNK3/MAPK10* [146]. *JNK1* and *JNK2* are widely expressed in most tissues, including the kidneys, while *JNK3* expression is primarily restricted to the central nervous system [142]. The sequence identity between JNK1 and JNK2 is 73% [146,147]. However, the ATP binding site is 98% conserved between the proteins, presenting a challenge to finding selective JNK inhibitors for research and therapeutics [148,149].

Alternative splicing adds complexity to the JNK pathway. Two independent splicing events produce at least four distinct isoforms of both JNK1 and JNK2 with differences at the C-terminal end and within the kinase domain [146]. The functional consequences of C-terminal splicing are not known. However, in the kinase domain, JNK containing exon 7b is more active in phosphorylating c-Jun than isoforms containing 7a [150]. JNK splicing regulation in the kidney has not been described, but it is possible that differential splicing could yield proteins with differing substrate specificities.

Activator protein-1 (AP-1) transcription factors are the most studied JNK substrates. JNK activates c-Jun by binding the transactivating domain and phosphorylating N-terminal serines 63 and 73 [141,151]. JNK similarly activates Activating Transcription Factor 2 (ATF2) [152]. This activation is critical for proliferation as fibroblasts expressing a mutant c-Jun that is incapable of JNK-mediated phosphorylation exhibited significantly reduced proliferation [153]. Interestingly, mice carrying this mutation developed normally, though they are smaller than controls. In contrast, c-Jun knockout mice die by embryonic day 13 with heart and liver malformations [154]. Overexpressing c-Jun in mice causes multi-organ fibrosis, including in the kidney and liver [155]. Although it is not discussed in the publication, c-Jun overexpression in kidney also caused cystic disease (see Figure 2E in [155]). Elevated levels of c-Jun have been detected in cancers including sarcomas [156] and Hodgkin lymphoma [157]. However, JNK and c-Jun/AP-1 can also have anti-tumor effects by participating in programmed cell death in response to DNA damage (158). Cell type and stimulus are contextual factors that influence the outcomes of JNK activation.

## JNK Involvement in Kidney Disease

JNK activation has been detected in many forms of kidney disease [159]. In animal models, JNK inhibition prior to ischemia-reperfusion or tubule obstruction reduces inflammation and fibrosis, and preserves kidney function [73–77]. Interestingly, acute kidney injury exacerbates polycystic kidney disease [69–72]. In chronic kidney insult, progressive interstitial fibrosis contributes to organ failure. JNK inhibition reduces profibrotic factors in the kidney [76,77]. Furthermore, researchers produced severe kidney fibrosis in mice by overexpressing the JNK target *c-Jun* [155]. Whether JNK contributes to fibrosis primarily through activation in tubule cells, interstitial cells, or a combination is yet to be determined.

## JNK Signaling in ADPKD

As with tumor growth, ADPKD is characterized by abnormally sustained proliferation. Elevated AP-1 components in cystic epithelium from ADPKD patients and mice suggest a pro-proliferative role for JNK/AP-1 signaling in ADPKD pathology [160]. To better understand the signaling pathways regulated by Pkd1, Arnould *et al.* targeted a construct containing Pkd1’s C-terminal intracellular tail to the cell membrane in human embryonic kidney 293T (HEK-293T) cells expressing an AP-1 luciferase reporter [161]. Expressing the Pkd1 construct induced 5-fold greater AP-1 reporter activity compared to a control construct. They further showed that JNK1 kinase activity was elevated in cells expressing the Pkd1 C-terminus, but surprisingly neither p38 nor p44/ERK1 were affected. Finally, they showed that Pkd1-induced AP-1 and JNK activity were blocked by expressing dominant-negative forms of the Rho GTPases, Cdc42 and Rac1 as well as a dominant-negative form of calcium-dependent PKC-alpha. The researchers hypothesized that high Pkd1 expression during renal development drives proliferation via Rac1/Cdc42 and PKCalpha. Under normal conditions, the reduction in Pkd1 levels that occur once development is complete would reduce the pathway activity and stop proliferation in mature tissues [161].

In a subsequent publication, the same authors showed that expressing full-length Pkd2 in HEK-293T cells robustly activated AP-1 and c-Jun reporters [162]. Pkd2-dependent activation was reduced by dominant-negative Cdc42, Rac1, and RhoA. Unlike Pkd1, which did not activate p38 or ERK, Pkd2 expression activated p38 in addition to JNK. Unique to Pkd2, AP-1 activation depended on calcium-independent PKC-epsilon and not PKC-alpha. Interested in the combined function of Pkd1 and Pkd2, the researchers co-expressed the C-terminal 112 base pairs of Pkd1 together with full length Pkd2. This resulted in higher AP-1 activity than with Pkd2 alone. The augmentation could be reversed by dominant-negative PKC-alpha or treatment with a calcium chelator. The authors concluded that Pkd1 and Pkd2 stimulate AP-1 activity in an additive manner via PKC-alpha and PKC-epsilon, respectively [162]. In their discussion, the authors speculated that Pkd1/Pkd2 induction of AP-1 activity might be important in directing proliferation and differentiation during nephrogenesis. However, they did not address how their findings apply to ADPKD, in which low or absent polycystin proteins promote proliferation in mature tubule cells. We now know that overexpressing Pkd1 or Pkd2 in mice induces a cystic phenotype [61,62]. While the mechanism is not known, it is possible that overexpression disrupts the polycystin complex, mimicking a loss-of-function mutation.

An independent study similarly found that AP-1 and JNK activity were increased in HEK-293T cells upon transfection with membrane-targeted Pkd1 constructs [163]. They additionally showed that dominant negative heterotrimeric G proteins abrogated the activation, while wild-type G protein expression augmented it. They hypothesized that Pkd1 regulated JNK activity by modulating the availability of heterotrimeric G proteins for downstream signaling. In a subsequent study, Pkd1-overexpressing MDCK cells were resistant to thrombin-induced JNK activation and c-Jun phosphorylation while cells lacking Pkd1 had increased JNK activation in response to thrombin [104].

While an immortalized epithelial cell line derived from an ADPKD patient exhibited reduced c-Jun phosphorylation compared to control cells [164], cyst-lining epithelium with *Pkd1* mutations in human and mouse kidneys expressed high levels of phosphorylated c-Jun and ATF2 [160]. These findings suggest that *in vivo* context is important for JNK/AP-1 activation in ADPKD. Although the mechanism by which Pkd1 and Pkd2 regulate JNK/AP-1 signaling *in vivo* is not known, cell culture studies showed that activation depends on Rho GTPases [161,162] or heterotrimeric G proteins [104,163,165].

The evidence linking Pkd1 and Pkd2 to JNK/AP-1 activity led us to ask what role JNK signaling plays in cyst formation and disease progression downstream of polycystin mutations [140]. To do this, we crossed mice carrying floxed alleles of *Pkd2* and *Jnk1, Jnk2* null alleles, and a tamoxifen-inducible Cre. The offspring, which segregated these four alleles, were treated with tamoxifen in the early post-natal period through maternal delivery. *Pkd2* loss led to rapid cyst formation along with increased JNK and c-Jun phosphorylation. Phospho c-Jun was concentrated in the nuclei of cells lining cysts and pre-cystic tubules, suggesting that it might drive cyst formation rather than occur as a response to tissue disruption. Mice segregating both *Pkd2* and *Jnk1*/*Jnk2* alleles showed significantly reduced cystic disease compared to mice only missing *Pkd2*. Whereas *Pkd2* mutant kidneys showed extensive cysts throughout the organ, *Pkd2*/*Jnk1*/*Jnk2* triple mutants retained cysts only at the cortical-medullary boundary and showed less proliferation and less fibrosis. While *Jnk1* and *Jnk2* are thought to be largely interchangeable, organ-specific functions have been observed. Our studies found that Jnk1 drove the protective effect, as the loss of *Jnk1* was almost as protective as *Jnk1* and *Jnk2* double loss. As the early postnatal delivery of tamoxifen produces rapid cystic disease progression, we sought to determine if JNK loss was protective in slow-progressing disease by treating mice with tamoxifen in early adulthood. The experiment had to be terminated before the kidneys became cystic because the loss of *Pkd2* caused serious liver cysts. However, the loss of JNK activity dramatically reduced the severity of the liver cysts suggesting that the protective effects of JNK loss extend beyond the early post-natal period and protect the liver.

## Prospective

Our finding that removal of JNK activity is protective against cystic disease suggests that drugs targeting this branch of MAPK signaling should be explored as therapeutic targets. Drugs active against JNK are in development as anti-cancer treatments and several candidates have been identified. For example, SP600125 [166] is a potent inhibitor of JNK with activity against numerous cancers [167]. With relevance to PKD, SP600125 is protective against ischemic/reperfusion injury of murine kidneys [168]. Other drugs include the small molecules JNK-in-8 [169] and CC-90001 (NCT02510937) as well as a TAT-JIP1 peptide inhibitor Brimapitide [170] that has been used to prevent the development of diabetes in mice [171]. The extensive functions of JNK in regulating cellular physiology may limit its effectiveness as a long-term drug. Therefore, upstream kinases such as MAP3Ks may be better targets with less adverse effects. Further work will be required to identify the MAP3K that functions between the polycystins and JNK. However, assuming that the specific MAP3K can be identified, there is precedence for developing specific MAP3K inhibitors that are effective and safe. These include the MLK2/MLK3 inhibitor URMC-099 [172] that has been shown to suppress murine non-alcoholic steatohepatitis [173] and shows neuroprotection in a murine model of Alzheimer’s disease [174,175]. The ASK1 inhibitor GS-444217 is effective for the treatment of diabetic kidney disease in rodents [176]. Selonsertib, which also inhibits ASK1, has been tested in clinical trials for diabetic kidney disease [177] and hepatic fibrosis in patients with non-alcoholic steatohepatitis [178,179].

## Abbreviations:

ADPKD: Autosomal Dominant Polycystic Kidney Disease
AP-1: Activator Protein-1
cAMP: Cyclic Adenosine Monophosphate
CDCA: Cilia-Dependent Cyst-Activating
CFTR: Cystic Fibrosis Transmembrane conductance Regulator
ESRD: End Stage Renal Disease
GPCR: G-protein Coupled Receptor
HEK-293T: Human Embryonic Kidney 293T
JNK: Jun N-terminal Kinase
MAP: Mitogen-Activated Protein
MAPK: Mitogen-Activated Protein Kinase
MAP2K: Mitogen-Activated Protein Kinase Kinase
MAP3K: Mitogen-Activated Protein Kinase Kinase Kinase
MDCK: Madin-Darby Canine Kidney
PKD: Polycystic Kidney Disease
